# Disruption of the ERLIN–TM6SF2–APOB complex destabilizes APOB and contributes to non-alcoholic fatty liver disease

**DOI:** 10.1371/journal.pgen.1008955

**Published:** 2020-08-10

**Authors:** Bo-Tao Li, Ming Sun, Yun-Feng Li, Ju-Qiong Wang, Zi-Mu Zhou, Bao-Liang Song, Jie Luo

**Affiliations:** Hubei Key Laboratory of Cell Homeostasis, College of Life Sciences, Wuhan University, Wuhan, China; University of Michigan, UNITED STATES

## Abstract

Non-alcoholic fatty liver disease (NAFLD) is a metabolic disorder characterized by excess lipid accumulation in the liver without significant consumption of alcohol. The transmembrane 6 superfamily member 2 (*TM6SF2*) E167K missense variant strongly associates with NAFLD in humans. The E167K mutation destabilizes TM6SF2, resulting in hepatic lipid accumulation and low serum lipid levels. However, the molecular mechanism by which TM6SF2 regulates lipid metabolism remains unclear. By using tandem affinity purification in combination with mass spectrometry, we found that apolipoprotein B (APOB), ER lipid raft protein (ERLIN) 1 and 2 were TM6SF2-interacting proteins. ERLINs and TM6SF2 mutually bound and stabilized each other. TM6SF2 bound and stabilized APOB via two luminal loops. ERLINs did not interact with APOB directly but still increased APOB stability through stabilizing TM6SF2. This APOB stabilization was hampered by the E167K mutation that reduced the protein expression of TM6SF2. In mice, knockout of *Tm6sf2* and knockdown of *Tm6sf2* or *Erlins* decreased hepatic APOB protein level, causing lipid accumulation in the liver and lowering lipid levels in the serum. We conclude that defective APOB stabilization, as a result of ERLINs or TM6SF2 deficiency or E167K mutation, is a key factor contributing to NAFLD.

## Author summary

Non-alcoholic fatty liver disease (NAFLD) is a very common liver disorder that occurs in people who do not drink too much alcohol. It initiates from extra fat storage in the liver and can advance to hepatitis, fibrosis, liver failure and liver cancer. NAFLD is often associated with other health problems such as obesity, diabetes, and hyperlipidemia. The *TM6SF2* gene variant is a strong risk factor for NAFLD in humans. However, the mechanism by which loss of TM6SF2 protein causes NAFLD is unclear. Here, we demonstrate that TM6SF2 forms a complex with ERLINs and APOB. ERLINs and TM6SF2 stabilize each other, and TM6SF2 stabilizes APOB. In mice, ablating the expression of ERLINs or TM6SF2 lowers APOB protein level, causing lipid accumulation in the liver while decreasing lipid levels in the blood. These phenotypes resemble the symptoms of NAFLD patients carrying TM6SF2 mutations. We conclude that TM6SF2 promotes APOB stability via complex formation and that defective APOB stabilization is one of the underlying causes of NAFLD.

## Introduction

Non-alcoholic fatty liver disease (NAFLD) is one of the most common liver disorders affecting 20–30% of adults worldwide and higher of certain ethnic groups [1]. It covers a wide degree of liver damage, including simple fat deposition (steatosis), inflammation (non-alcoholic steatohepatitis), and scarring (fibrosis and cirrhosis) [2–4]. NAFLD increases the risks of hepatocellular carcinoma [5, 6], and is often associated with comorbidities such as obesity, insulin resistance, hypertension, and hyperlipidemia [7]. However, no consensus has been reached on the causal relationship between NAFLD and the metabolic complications [8, 9].

Both environmental and genetic factors contribute to the pathogenesis and progression of NAFLD. By using genome-wide association studies, multiple NAFLD-related risk variants have been identified, among which include rs58542926 in the transmembrane 6 superfamily member 2 (*TM6SF2*) gene that causes a nonsynonymous glutamate-to-lysine substitution at the amino acid residue 167 (E167K) [10, 11]. TM6SF2 is a transmembrane protein mainly localized in the endoplasmic reticulum (ER), ER-Golgi intermediate compartment and Golgi of hepatocytes and enterocytes [12, 13]. It critically regulates lipid metabolism in the liver [11]. The E167K mutation markedly reduces TM6SF2 protein level through enhancing its turnover rate [11, 14], and the *TM6SF2* E167K carriers exhibit high hepatic triglyceride (TG) content but low plasma low-density lipoprotein cholesterol (LDL-C) levels with improved cardiovascular outcomes [10, 11]. In mice, knockout or knockdown of *Tm6sf2* recapitulates the human NAFLD phenotypes [10, 11, 13, 15], whereas liver-specific overexpression of *TM6SF2* elevates plasma total cholesterol (TC) and LDL-C levels with, however, mixed results on hepatic TG content [10, 15]. TM6SF2 is shown to facilitate the secretion of TG-rich very low-density lipoproteins (VLDLs), and to a lesser extent, that of apolipoprotein B (APOB) in human hepatoma cell lines [12]. The hepatocyte spheroids derived from the *TM6SF2* E167K carriers are less capable of secreting APOB than those from wild-type (WT) controls [16]. However, another study proposes that TM6SF2 is required for VLDL assembly but not APOB-containing lipoprotein secretion [13]. The exact roles of TM6SF2 in APOB metabolism and NAFLD remain unclear.

ER lipid raft protein (ERLIN, also known as stomatin-prohibitin-flotillin-HflC/K domain-containing protein) 1 is an ER-resident, single-pass transmembrane glycoprotein that forms a heteroligomeric complex with its closely related ERLIN2 towards the ER lumen to mediate degradation of 1,4,5-trisphosphate receptors and 3-hydroxy-3-methylglutaryl CoA-reductase, the latter of which is the rate-limiting enzyme in the cholesterol biosynthetic pathway [17–19]. ERLIN1 and ERLIN2 also suppress cholesterol production by blocking the export of sterol regulatory element-binding proteins from the ER to the Golgi under high cholesterol conditions [20]. Interestingly, a missense mutation in *ERLIN1* is recently suggested to confer protection against fatty liver and hepatic inflammation [21, 22]. How ERLINs are implicated in NAFLD is yet to be established.

In this study, we employed tandem affinity purification (TAP) coupled with mass spectrometry to identify TM6SF2-interacting proteins. We found that TM6SF2, ERLIN1 and ERLIN2 formed a protein complex with APOB, which is mainly produced by the intestine and liver [23]. TM6SF2 could stabilize and be stabilized by ERLIN1 and ERLIN2. The two luminal loops of TM6SF2 were critical for APOB48 binding and stabilization. The E167K mutation hampered APOB48 stabilization by reducing the expression of TM6SF2. Whole-body knockout of *Tm6sf2* and adeno-associated virus serotype 8 (AAV8)-mediated knockdown of *Tm6sf2* or *Erlins* decreased hepatic APOB expression, increased TC and TG levels in the liver and reduced lipids in the serum. We conclude that TM6SF2 promotes APOB stability via complex formation and that defective APOB stabilization is one of the underlying causes of NAFLD.

## Results

### Identification of APOB and ERLINs as TM6SF2-associated proteins regulating lipid droplet (LD) content in the cell

We first sought to identify the proteins that interact with TM6SF2. Since TM6SF2 is a multi-pass transmembrane protein, we employed a tandem affinity purification strategy to improve purification specificity. A construct containing the mouse *Tm6sf2* gene followed by the sequences encoding a 3×FLAG epitope tag, a tobacco etch virus (TEV) cleavage site and a 2×Protein A epitope tag was generated (TM6SF2-TAP; S1A Fig) and transfected into CRL1601 cells, a rat hepatoma cell line. Consistent with the previous results [12], cells transiently expressing TM6SF2-TAP had less LDs compared with neighboring non-expressing ones (S1B Fig), suggesting that the TM6SF2-TAP fusion protein is functional. To obtain a single cell clone stably expressing TM6SF2-TAP, transiently transfected cells were subjected to about one-week antibiotic selection and surviving colonies were isolated and expanded. Compared with the parental CRL1601 cells, the stable clone (CRL1601/TM6SF2-TAP) had much higher expression of TM6SF2 (S1C Fig). We next pulled down TM6SF2 and the associated proteins from the CRL1601/TM6SF2-TAP stable cells using the IgG-coupled agarose. After digesting with the TEV protease, supernatants were further immunoprecipitated using the anti-FLAG beads followed by elution using the FLAG peptides (S1D Fig). The eluent was resolved by sodium dodecyl sulfate–polyacrylamide gel electrophoresis (S1E Fig) and analyzed by tandem mass spectrometry. The top 14 proteins including TM6SF2 were subjected to further investigation (S1F Fig).

**Fig 1:**
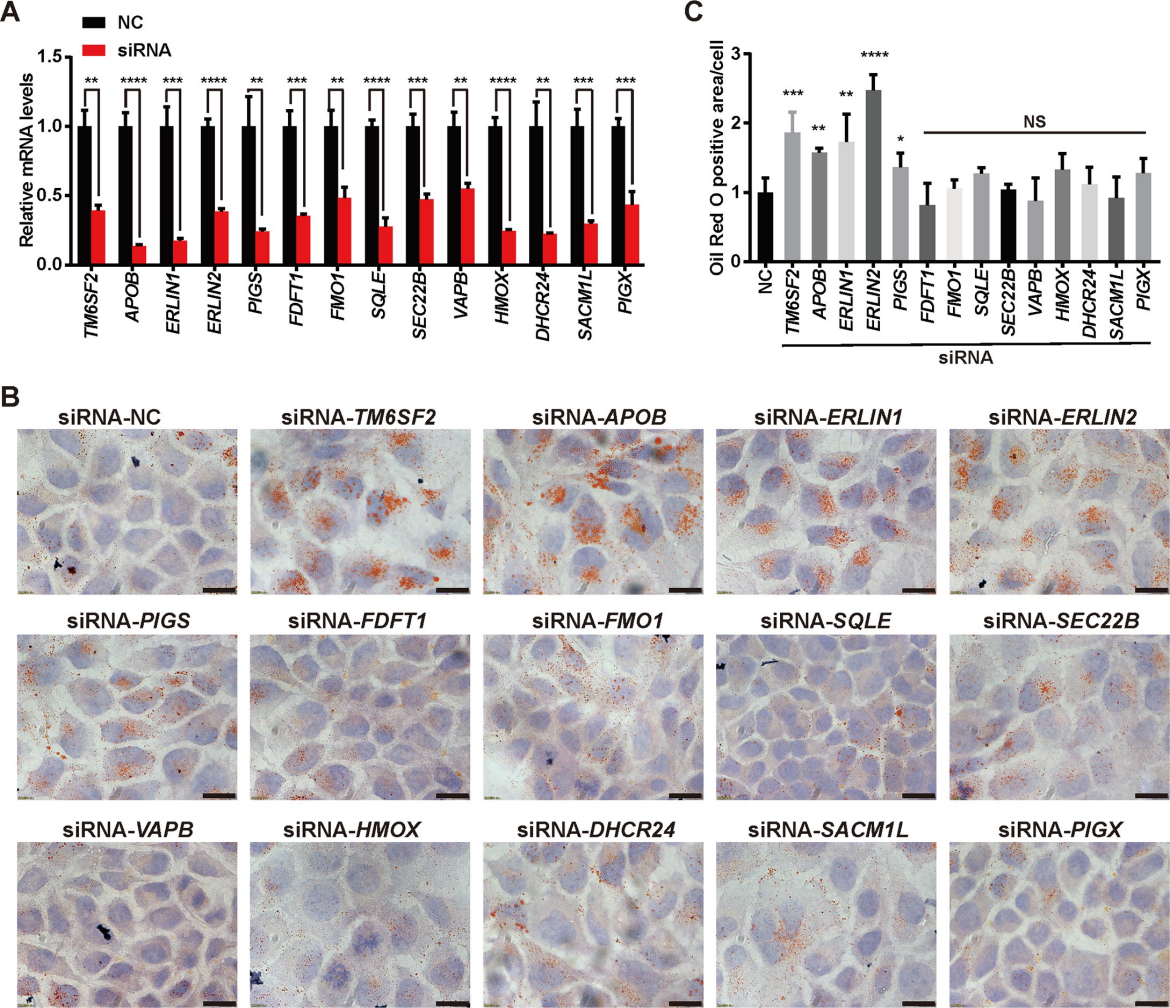
Knockdown of *TM6SF2*, *APOB*, *ERLIN1* or *ERLIN2* increases cellular LD content. Huh7 cells were transfected with the indicated siRNAs. After 48 h, cells were harvested for quantitative real-time PCR (A) or Oil Red O staining (B-C). (A) Knockdown efficiency of the indicated siRNA duplexes. Data are presented as mean±SD (n = 3 independent experiments). Student’s *t*-test. **P<0.01, ***P<0.001, ****P<0.0001. (B) Representative Oil Red O staining showing LDs in Huh7 cells transfected with the indicated siRNAs. NC, negative control. Scale bars, 20 μm. (C) Quantification of Oil Red O-stained LD area per cell number in (B). Data are normalized to control cells and presented as mean±SD (n = 2 independent experiments; 30–40 cells per experiment). Student’s *t*-test. *P<0.05, **P<0.01, ***P<0.001, ****P<0.0001, NS, no significance.

We next knocked down each of the 14 candidate genes using RNA interference (Fig 1A) and examined the LD content in Huh7 cells, a human hepatocellular carcinoma cell line. TM6SF2 absence was used as a positive control. Robust LD accumulation was detected upon *APOB*, *ERLIN1* or *ERLIN2* deficiency (Fig 1B and 1C). Knockdown of GPI transamidase component PIG-S (*PIGS*) produced a modest phenotype, whereas of others was ineffective (Fig 1B and 1C).

**Fig 2:**
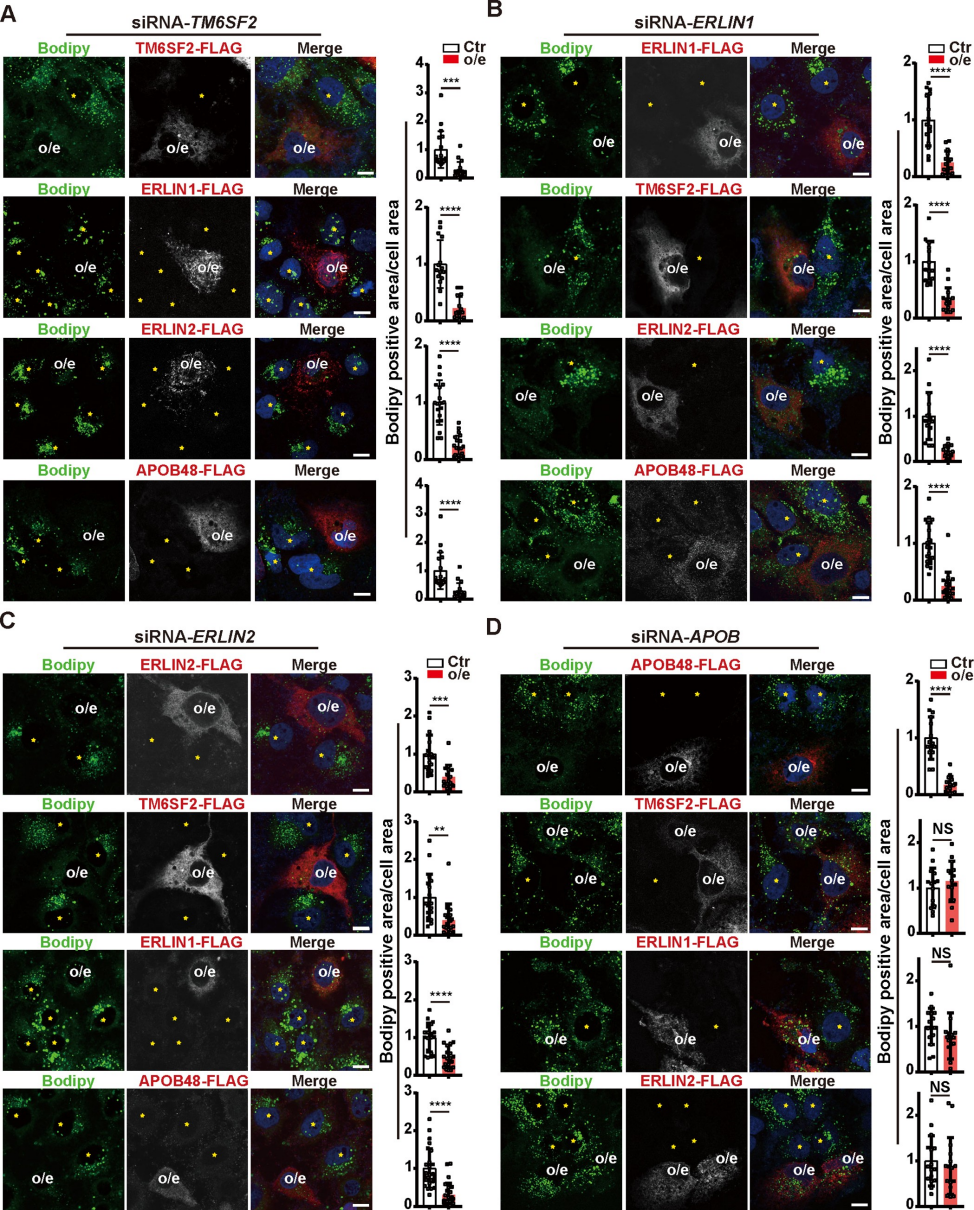
TM6SF2, ERLIN1 and ERLIN2 function upstream of APOB. Huh7 cells were transfected with siRNAs targeting *TM6SF2* (A), *ERLIN1* (B), *ERLIN2* (C) or *APOB* (D) and the indicated plasmids, among which included an siRNA-resistant plasmid that carries a synonymous mutation in the knocked down gene to exclude siRNA off-target effects. After 48 h, cells were fixed, with LDs stained with Bodipy (green) and the transiently expressed proteins with the anti-FLAG antibody (Red). Nuclei were counterstained with DAPI (blue). Scale bars, 10 μm. Cells overexpressing the transfected protein (o/e) and neighboring un-transfected ones (yellow stars) are indicated. Quantification of Bodipy-stained area per cell area is shown on the right. Data are normalized to control cells and presented as mean±SD (n = 2 independent experiments; at least 15 cells per experiment). Student’s *t*-test. **P<0.01, ***P<0.001, ****P<0.0001, NS, no significance.

We next evaluated the epistatic relationship between TM6SF2, ERLIN1, ERLIN2 and APOB in regulating LD secretion. Huh7 cells were co-transfected with siRNAs targeting one gene and plasmids encoding each of the other genes (Fig 2). An siRNA-resistant plasmid that carries a synonymous mutation in the gene of interest was also used to exclude the off-target effects of siRNAs (Fig 2A–2D, first row). In *TM6SF2*-knockdown cells, LD buildup was effectively alleviated by overexpression of ERLIN1, ERLIN2 or APOB48 (Fig 2A). The ectopically expressed TM6SF2, ERLIN1, ERLIN2 and APOB48 also reduced the LD areas in *ERLIN1*- or *ERLIN2*-knockdown cells (Fig 2B and 2C). Of note, neither TM6SF2 nor ERLINs was able to revert LD accumulation in *APOB*-knockdown cells, contrasting to the rescue effect by APOB48 (Fig 2D). These results suggest that TM6SF2 and ERLINs function upstream of APOB to regulate LD metabolism.

### TM6SF2 forms a protein complex with ERLINs and APOB

**Fig 3:**
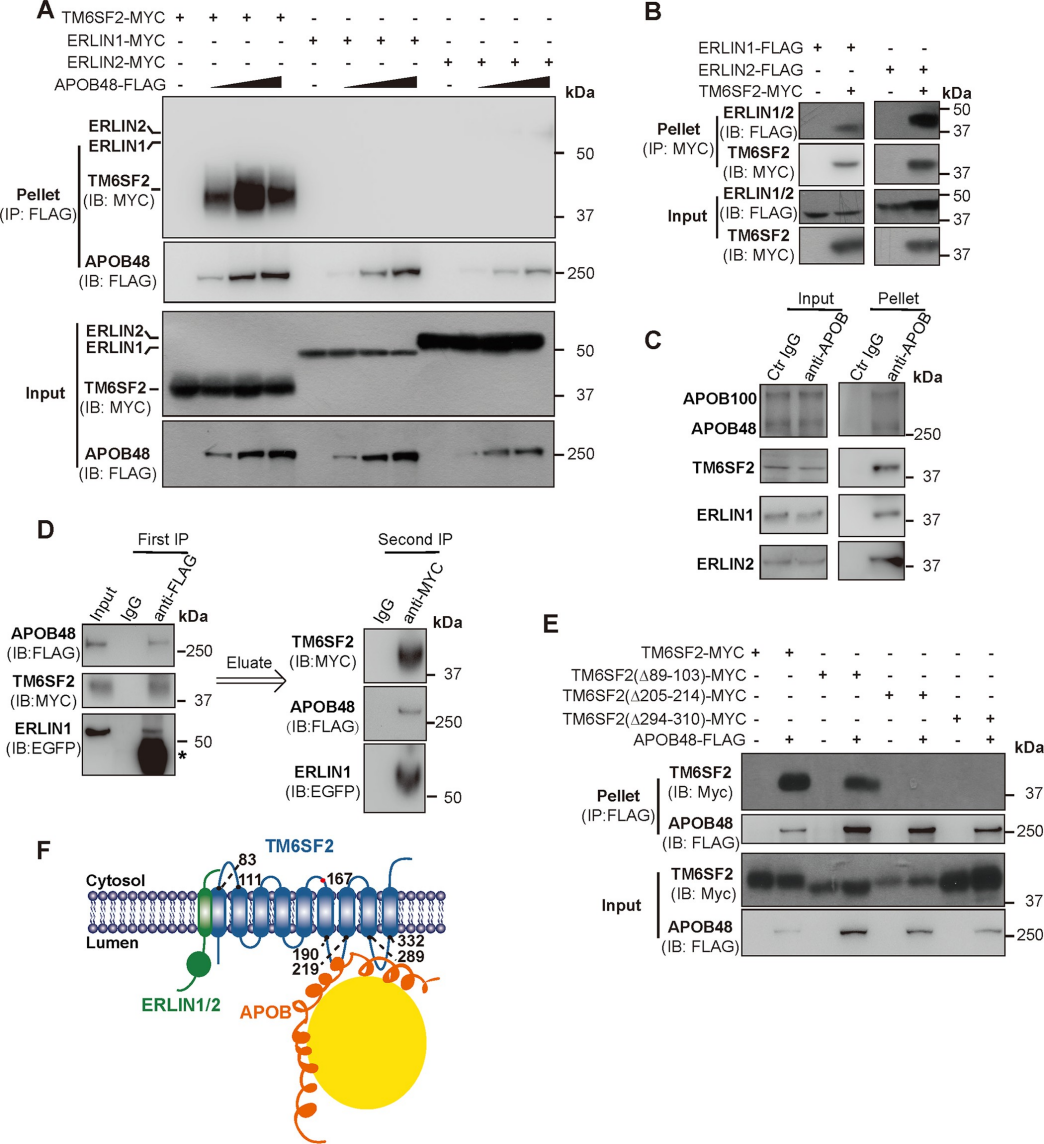
TM6SF2 interacts with ERLIN1, ERLIN2 and APOB48. (A) APOB48 binds TM6SF2 but not ERLIN1 or ERLIN2. Huh7 cells were transfected as indicated. After 48 h, cells were harvested, and APOB48 was immunoprecipitated using the anti-FLAG coupled agarose followed by probing for the indicated antibodies. (B) TM6SF2 binds ERLIN1 and ERLIN2. Huh7 cells were transfected as indicated. After 48 h, cells were harvested, and TM6SF2 was immunoprecipitated using the anti-MYC coupled agarose followed by probing for the indicated antibodies. (C) Endogenous APOB, TM6SF2 and ERLINs bind each other. APOBs were immunoprecipitated from CRL1601 lysates using protein A/G beads coupled with the anti-APOB antibody and analyzed by immunoblotting with the anti-TM6SF2, anti-ERLIN1 and anti-ERLIN2 antibodies. Protein A/G beads (Ctr IgG) were used as a control. (D) Sequential immunoprecipitation analysis of Huh7 cells transfected as indicated. Lysates were first immunoprecipitated using the anti-FLAG beads and analyzed by immunoblotting with the indicated antibodies. Proteins were then eluted with the 3×FLAG peptide and subjected to a secondary immunoprecipitation followed by immunoblotting analysis. Asterisk indicates non-specific band. (E) APOB48 binds TM6SF2 via two large luminal loops. Huh7 cells were transfected as indicated. After 48 h, cells were harvested, and APOB48 was immunoprecipitated using the anti-FLAG coupled agarose followed by immunoblotting with the indicated antibodies. (F) Schematic showing the ERLIN1/2–TM6SF2–APOB complex.

To investigate whether these proteins physically interact with each other, we immunoprecipitated APOB48 from Huh7 cells co-transfected with the plasmids encoding FLAG-tagged APOB48 and Myc-tagged TM6SF2 or ERLIN1 or ERLIN2. A significant amount of TM6SF2 was detected in the APOB48 pellet (Fig 3A). ERLIN1 and ERLIN2 were efficiently co-immunoprecipitated with TM6SF2 (Fig 3B) but not with APOB48 (Fig 3A).

We next immunoprecipitated endogenous APOB100 and APOB48 from CRL1601 cells using protein A/G beads coupled with the anti-APOB antibody. Fig 3C showed that TM6SF2, ERLIN1 and ERLIN2 were present in the pellets. To rigorously demonstrate that TM6SF2, ERLINs and APOB form a protein complex, we performed a sequential immunoprecipitation experiment using Huh7 cells transfected with the plasmids encoding FLAG-tagged APOB48, Myc-tagged TM6SF2 and EGFP-tagged ERLIN1. The first immunoprecipitation was performed using the anti-FLAG beads. After elution with the 3×FLAG peptide, eluent was subjected to a secondary immunoprecipitation with the anti-Myc beads. TM6SF2, ERLIN1 and APOB48 were detected in the second pellet (Fig 3D), suggesting that these proteins are in fact in the same complex.

To further determine the specific domains mediating TM6SF2–APOB48 interaction, we generated TM6SF2 truncations lacking the large cytosolic loop (Δ89–103) or two luminal loops (Δ205–214 and Δ294–310). Compared with the WT protein, TM6SF2 (Δ89–103) still, albeit to a lesser extent, bound to APOB48, whereas TM6SF2 (Δ205–214) or TM6SF2 (Δ294–310) failed to do so (Fig 3E). These results suggest that TM6SF2 can form a protein complex with APOB48 via two luminal loops as well as with ERLIN1 and ERLIN2, which, however, do not interact with APOB48 (Fig 3F).

**Fig 4:**
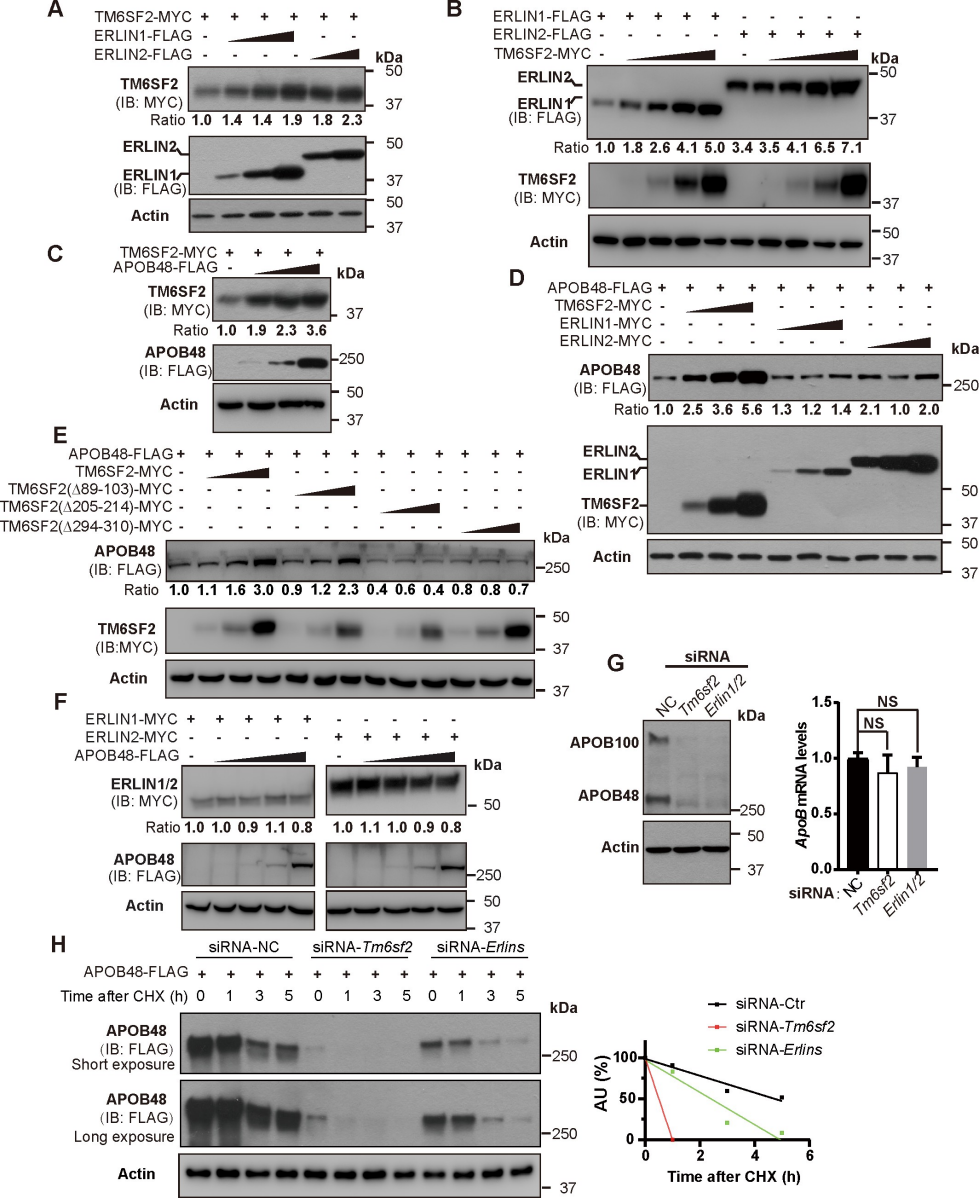
Stabilization of the ERLINs–TM6SF2–APOB complex. (A-F) Huh7 cells were transfected as indicated. After 48 h, cells were harvested for immunoblotting analysis. The densitometry of indicated protein in the first row of each panel was normalized to that of corresponding actin. The normalized value in the control group is defined as 1. (A-B) TM6SF2 is stabilized by increasing concentrations of ERLINs (A) and vice versa (B). (C) TM6SF2 is stabilized by increasing concentrations of APOB48. (D) APOB48 is markedly stabilized by increasing concentrations of TM6SF2 but not ERLINs. (E) TM6SF2 stabilizes APOB48 via two large luminal loops. (F) APOB48 cannot stabilize ERLINs. (G) CRL1601 cells were transfected with the indicated siRNAs and harvested for immunoblot and quantitative real-time PCR analysis. NC, negative control. Data are normalized to control cells and presented as mean±SD (n = 3 independent experiments). One-way ANOVA with Dunnett’s post hoc test. NS, no significance. (H) CRL1601 cells were transfected with FLAG-tagged APOB and the indicated siRNAs. Cells were then treated with 100 μM cycloheximide (CHX) for indicated periods and harvested for immunoblotting. Densitometric analysis of APOB48 levels was on the right. AU, arbitrary unit.

We next set out to determine how each component of the ERLINs–TM6SF2–APOB48 protein complex affects the stability of one another. ERLIN1 and ERLIN2 increased the protein level of TM6SF2 in a dose-dependent manner and vice versa (Fig 4A and 4B). TM6SF2 and APOB48 could stabilize each other (Fig 4C and 4D). The two luminal loops that mediate TM6SF2–APOB48 interaction were also critical for APOB48 stabilization by TM6SF2 (Fig 4E). ERLINs and APOB48 had little, if any, effects on one another (Fig 4D and 4F). Knockdown of *Tm6sf2* or *Erlins* nearly eliminated the endogenous APOB100 and APOB48 levels without, however, interfering *APOB* mRNA abundance (Fig 4G). Cycloheximide chase analysis of CRL1601 cells transfected with FLAG-tagged APOB48 together with control siRNA or siRNAs targeting *Tm6sf2* or *Erlins* showed that degradation of APOB48 was accelerated in *Tm6sf2*- or *Erlins*-depleted cells (Fig 4H), excluding the possibility that decreases in APOB protein levels might result from reduced APOB biosynthesis.

**Fig 5:**
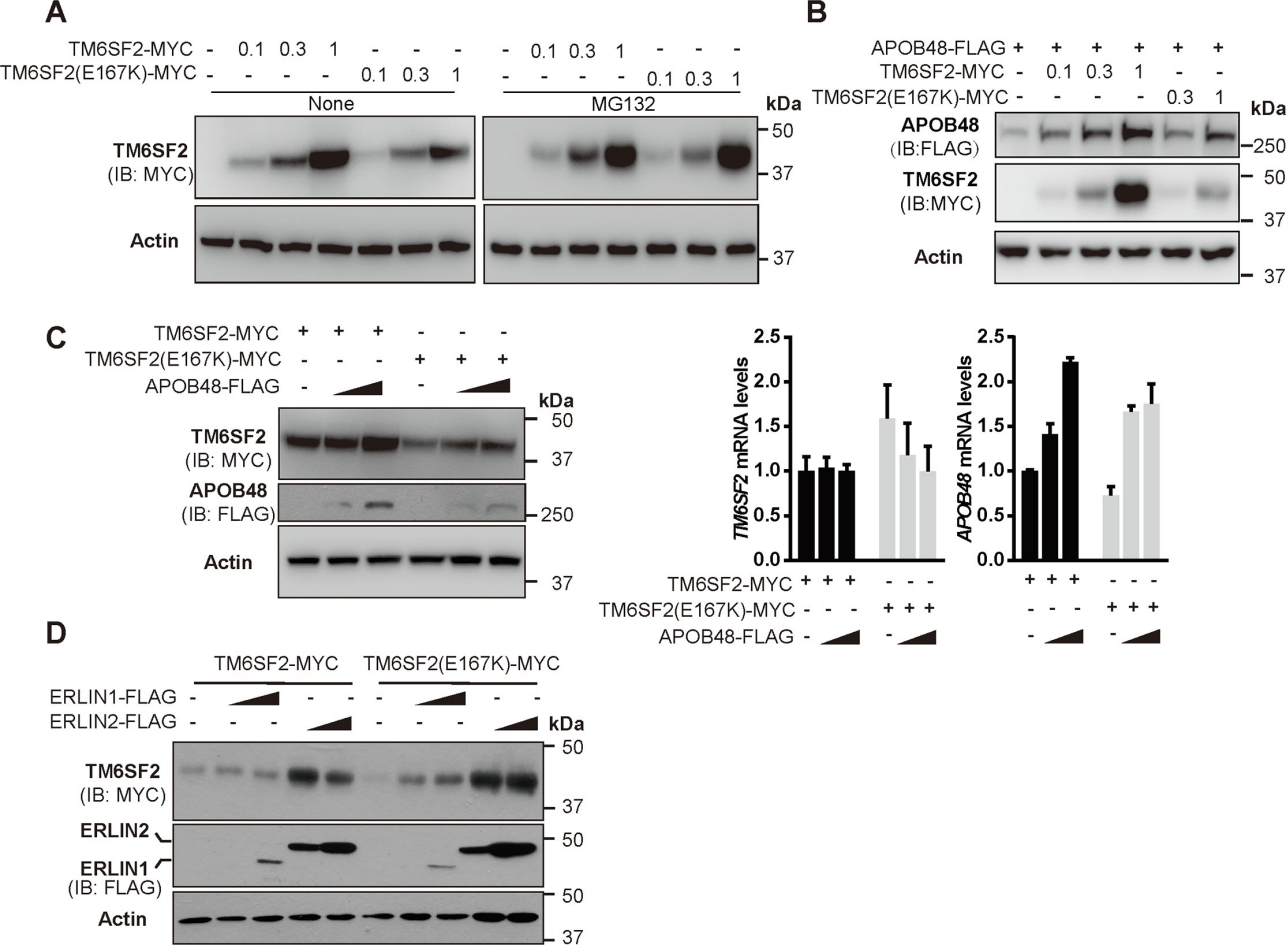
The E167K mutation reduces TM6SF2 protein level and destabilizes APOB. (A-B) Huh7 cells were transfected with increasing concentrations of plasmids expressing WT and E167K mutant form of TM6SF2. After 48 h, cells were treated without or with 10 μM MG132 for 2 h and then harvested for immunoblotting. (C) Huh7 cells were transfected as indicated. After 48 h, cells were harvested for immunoblotting and quantitative real-time PCR. Data are normalized to cells transfected with TM6SF2 only and presented as mean±SD (n = 3 independent experiments). (D) Huh7 cells were transfected as indicated and harvested 48 h later for immunoblotting.

We also examined the effects of TM6SF2 E167K mutation on APOB48 stability. In line with the previous findings that TM6SF2 (E167K) is unstable [11, 15], we observed less protein level of the E167K mutant than that of the WT form transfected in equal amounts (Fig 5A). The proteasome inhibitor MG132 effectively reverted the expression defect caused by E167K mutation (Fig 5A). Compared with the WT protein, the E167K mutant was less potent in stabilizing APOB48 (Fig 5B). However, the E167K mutation did not affect the stabilizing effects of APOB (Fig 5C) and ERLINs (Fig 5D) on TM6SF2.

Together, these results suggest that APOB48 can be stabilized by directly associated TM6SF2 as well as by ERLINs via TM6SF2. Such stabilizing effects on APOB48 are attenuated by E167K mutation that decreases protein expression of TM6SF2.

### Deficiency of *Tm6sf2* or *Erlins* causes LD accumulation in mouse liver

**Fig 6:**
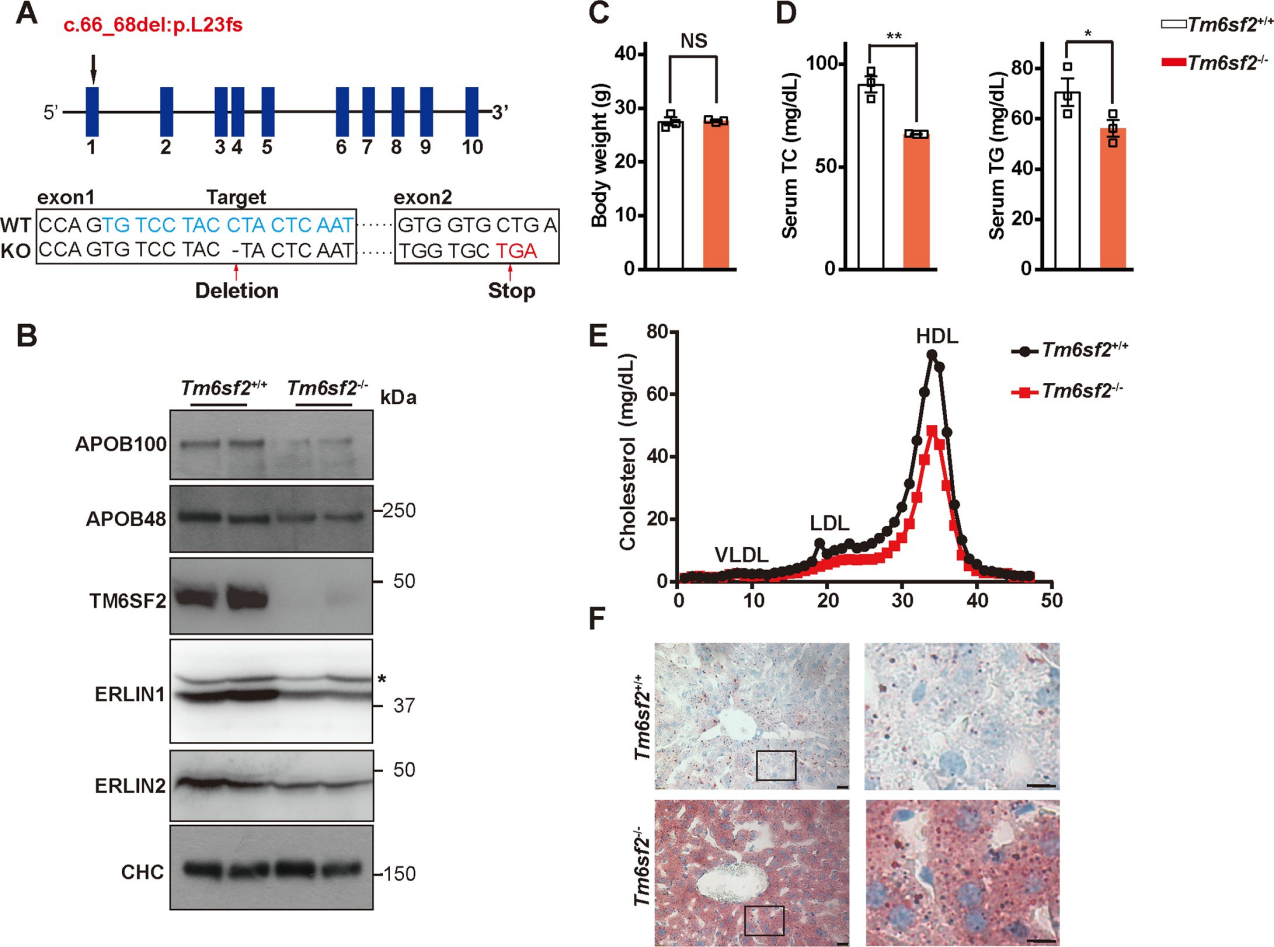
Generation and characterization of whole-body *Tm6sf2* knockout mice. (A) Genomic structure of mouse *Tm6sf2* gene. The spacer region for TALEN is in blue. TALEN-based genome editing was used to introduce 1 bp deletion at the site 67 in exon 1, which results in an L23 frameshift (fs) mutation and premature translation termination that occurs in exon 2. (B-F) Thirteen-week-old male WT (*Tm6sf*^*+/+*^) and *Tm6sf2* knockout (*Tm6sf2*^*-/-*^) mice (n = 3 per group) maintained on a chow diet were used. Mice were fasted for 4 h and sacrificed for various assays. (B) Representative blots showing the expression of APOB, TM6SF2, ERLIN1 and ERLIN2 in the livers of *Tm6sf2*^*+/+*^ and *Tm6sf2*^*-/-*^ mice. Clathrin heavy chain (CHC) was used as a loading control. Asterisk indicates non-specific bands. (C) Body weight. (D) Serum total cholesterol (TC) and triglyceride (TG) levels. Data are presented as mean±SD. Student’s *t*-test. *P<0.05, **P<0.01, NS, no significance. (E) Cholesterol distribution in serum lipoprotein fractions determined by fast-performance liquid chromatography. (F) Oil Red O staining of liver sections from *Tm6sf2*^*+/+*^ and *Tm6sf2*^*-/-*^ mice. Boxed areas are shown at a higher magnification on the right. Scale bars, 20 μm (main), 10 μm (inset).

To gain insight into the *in vivo* function of TM6SF2, we generated *Tm6sf2* knockout mice (*Tm6sf2*^-/-^) by introducing a deletion at the coding nucleotide 67 that causes an L23 frameshift mutation in *Tm6sf2* (c.66_68del:p.L23fs) using the TALEN strategy (Fig 6A). Mice were fed a standard chow diet for 13 weeks. Consistent with the results in Fig 4G, the protein levels of APOB100, APOB48, ERLIN1 and ERLIN2 were substantially lowered in the *Tm6sf2*^-/-^ livers (Fig 6B). Loss of TM6SF2 did not alter body weight (Fig 6C) but significantly reduced serum levels of TC and TG (Fig 6D) as seen in human *TM6SF2* E167K carriers. Cholesterol levels in low- and high-density lipoprotein fractions were also decreased in *Tm6sf2*^-/-^ mice as measured by fast-performance liquid chromatography (Fig 6E). Moreover, *Tm6sf2*^*-/-*^ mice had apparent lipid accumulation in the liver as revealed by Oil Red O staining (Fig 6F).

**Fig 7:**
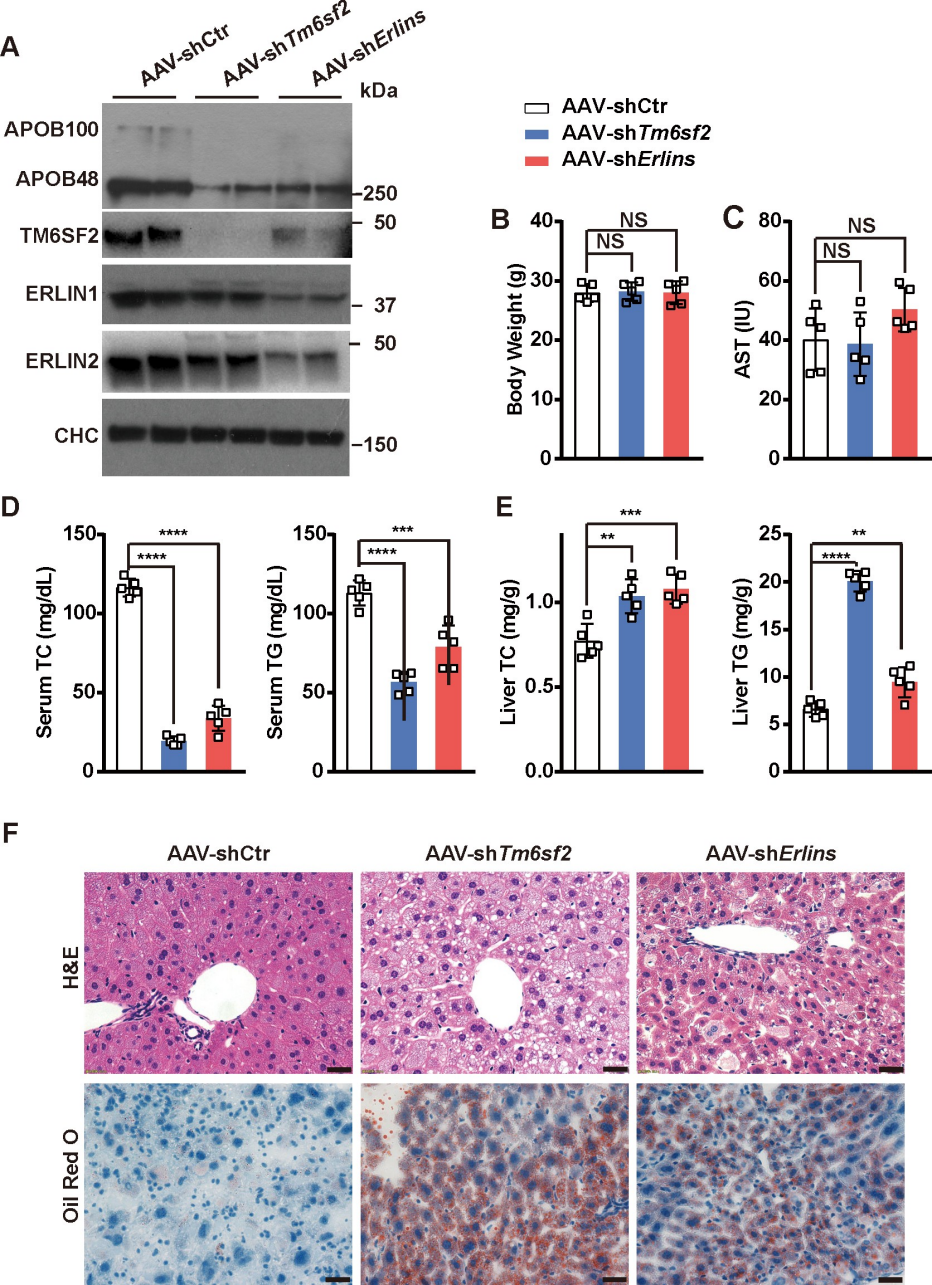
AAV8-mediated knockdown of *Tm6sf2* or *Erlins* induces lipid accumulation in the liver and reduces lipid levels in the serum. Eight-week-old BALB/c male mice on a chow diet were intravenously injected with AAV8-expressing control (Ctr) shRNA, or *Tm6sf2*- or *Erlins*-specific shRNAs (n = 5 per group) and subjected to various analyses 2 weeks later. Mice were fasted for 4 h and sacrificed for various assays. (A) Representative blots showing the expression of APOB, TM6SF2, ERLIN1 and ERLIN2 in the livers of mice receiving indicated injections. CHC was used as a loading control. (B) Body weight. (C) Serum aspartate aminotransferase (AST) levels. (D) Serum TC and TG levels. (E) Liver TC and TG levels. Data are presented as mean±SD. One-way ANOVA with Dunnett’s post hoc test. **P<0.01, ***P<0.001, ****P<0.0001, NS, no significance. (F) H&E (*Top*) and Oil Red O (*Bottom*) staining of liver sections from mice receiving indicated injections. Scale bars, 30 μm.

To investigate the *in vivo* functions of ERLINs in APOB and lipid metabolism, we selectively knocked down *Tm6sf2* or both *Erlin1* and *Erlin2* (*Erlins*) in mouse liver using AAV8-expressing short hairpin RNAs (shRNAs). AAV8 administration via tail vein injection has been widely used for efficient gene delivery to the mouse liver [11, 24, 25]. AAV8 expressing control shRNA (AAV-shCtr) was used as a control. In line with previous results [11], the *Tm6sf2* mRNA abundance in the liver but not adipose tissues or small intestine was profoundly abolished by AAV-sh*Tm6sf2* (S2 Fig). Mice receiving AAV-sh*Tm6sf2* or AAV-sh*Erlins* injections showed markedly reduced APOB protein levels in the liver (Fig 7A). Silencing of *Tm6sf2* also diminished ERLIN protein expression and vice versa (Fig 7A). Neither body weight (Fig 7B) nor aspartate aminotransferase level (Fig 7C) was altered upon *Tm6sf2* or *Erlins* deficiency, suggesting no obvious toxicity after knocking down *Tm6sf2* or *Erlins*. The serum TC and TG were substantially decreased whereas the hepatic TC and TG were significantly increased in *Tm6sf2*- and *Erlins*-knockdown mice (Fig 7D and 7E). Similar to those lacking *Tm6sf2* (Fig 6F), hepatic lipid accumulation was evident in mice injected with AAV-sh*Tm6sf2* or AAV-sh*Erlins* (Fig 7F). Together, these results suggest that both TM6SF2 and ERLINs contribute to APOB stabilization *in vivo*, and that depletion of TM6SF2 or ERLINs causes lipid accumulation in the liver and lowers lipid levels in the blood.

## Discussion

NAFLD is a multifactorial disease that, in the context of metabolism, results from increased lipid biosynthesis, excessive fat uptake, reduced fatty acid oxidation or decreased lipid secretion. In addition to the established risk factors such as age, body mass index and gender, genetic variants in *TM6SF2*, patatin-like phospholipase domain containing 3 (PNPLA3), glucokinase gene regulator, and many others also confer susceptibility to NAFLD [11, 26–31]. The effects of TM6SF2, PNPLA3 and glucokinase gene regulator on NAFLD are additive in some individuals [32, 33] and can be augmented by obesity [34]. Interestingly, TM6SF2 is also a critical determinant of plasma lipid levels, which are lower in human TM6SF2(E167K) carriers [10, 11, 33, 35]. How TM6SF2 underlies such a clinical paradox is poorly understood.

The *TM6SF2* E167K missense mutation has been reported to reduce plasma APOB levels in human populations [35, 36], at least in part, by decreasing its secretion [16]. *TM6SF2* silencing also inhibits APOB secretion in two human hepatoma cell lines [12]. However, a recent study reports impaired VLDL lipidation but normal APOB secretion in *Tm6sf2*^-/-^ mice [13]. We hereby show that ERLIN1 and ERLIN2 are the interacting partners of TM6SF2, which also binds APOB via two luminal loops. The ERLINs–TM6SF2–APOB complex promotes APOB stabilization. Deficiency of TM6SF2 and ERLINs decreases APOB protein levels and induces fat accumulation in the liver, as well as reduces lipids in the blood. The E167K mutation reduces TM6SF2 levels and, therefore, cannot stabilize APOB as the WT form does. This destabilization of APOB may account for defective lipidation or secretion of APOB-containing lipoprotein particles as reported in other studies. Moreover, we show that, while ERLINs do not bind APOB directly (Fig 3A) and ERLIN overexpression has no effect on APOB level (Fig 4D), depletion of ERLINs is sufficient to eliminate APOB expression in an extent similar to that of TM6SF2 (Fig 4G). These results suggest that the optimal stabilizing activity may require a specific ratio of APOB, TM6SF2 and ERLINs, and a disruption of the stoichiometric balance can compromise the complex function. This may also explain a recent result that APOB secretion is attenuated in mice with AAV8-mediated overexpression of *Tm6sf2* [14].

Notably, we found that hepatic APOB100 and APOB48 proteins were decreased in mice with global deficiency or hepatic knockdown of *Tm6sf2* (Figs 6B and 7A), contrasting to the unaltered APOB levels previously found in *Tm6sf2*^-/-^ mice [13]. We hypothesize such a discrepancy may be attributed to the procedure difference between Smagris’ work and ours, where they fasted mice from 7:00 am to 11:00 am (Procedure #1) but we did from 10:00 am to 2:00 pm (Procedure #2). When these two paradigms were compared side by side, slight but not significant decreases in APOB levels were detected in *Tm6sf2*^*-/-*^ mice under Procedure #1, and more prominent under Procedure #2 (S3A Fig). Under both conditions, TC and TG were increased in the liver and decreased in serum upon *Tm6sf2* deficiency, respectively (S3B and S3C Fig).

Our results show that reduced APOB stabilization and secretion can enhance lipid accumulation in the liver and may underlie a subclass of NAFLD. Very recently, APOE destabilization caused by PNPLA7 deficiency was reported to inhibit hepatic VLDL secretion and exacerbate fatty liver in *db*/*db* mice [37]. PNPLA7, akin to TM6SF2, directly binds and stabilizes APOE, and knockdown of PNPLA7 increases ubiquitination of APOE and that of APOB in an APOE-dependent manner [37]. Whether formation of the ERLINs–TM6SF2–APOB complex may inhibit ubiquitination and degradation of APOB is worthy of further investigation. Nevertheless, these results suggest that increasing hepatic TG clearance by promoting APOB and VLDL secretion may serve as a promising strategy for the treatment of NAFLD.

Given that deficiency of TM6SF2 impairs TG transportation from the liver to circulation, one might assume high-fat diet (HFD) challenge could increase hepatic lipid accumulation and aggravate hepatic steatosis. A study by Fan and colleagues showed that liver TG content was markedly elevated by 12-week HFD feeding, but the increases were indistinguishable in WT and *Tm6sf2*^*-/-*^ mice [15]. These results may be attributable to the attenuated TM6SF2 expression following HFD [15], which mitigates the impact of TM6SF2 deficiency on hepatic lipid metabolism. In addition, under 12-week HFD feeding, their WT mice already exhibited drastic LD accumulation in the liver, suggesting that the feeding period might be too long to distinguish the difference between WT and *Tm6sf2*^*-/-*^ mice. In fact, Kozlitina et al. used a high-sucrose diet and found much more LDs in *Tm6sf2*-knockdown mouse liver [11]. We also observed dramatic lipid accumulation in *Tm6sf2*-knockout mouse liver under chow diet (Fig 6F). The effects of different diets on NAFLD under different genetic backgrounds need further investigation.

It has been demonstrated that ectopically expressed TM6SF2 increases cholesterol biosynthesis via activation of 7-dehydrocholesterol reductase, the last enzyme in the Kandutsch-Russell pathway [15, 38]. However, mice lacking *Tm6sf2* show no changes in the cholesterol content or cholesterogenic gene expression in the liver [13, 15], but demonstrate increased hepatic cholesteryl esters as human E167K carriers [13, 39]. Although the role of TM6SF2 in cholesterol metabolism under physiological conditions warrants further characterization, ERLINs can reduce cholesterol production by inhibiting SREBP-2 processing and promoting 3-hydroxy-3-methylglutaryl CoA-reductase degradation [17, 18, 40]. As ERLINs define the lipid-raft-like microdomains in the ER [15], we hypothesize that ERLINs recruit TM6SF2 and the associated APOB to the sterol-rich ER membrane microdomains, where newly synthesized cholesteryl esters and TG are added to nascent APOB-containing lipoproteins.

Taken together, we show that ERLIN1 and ERLIN2 are TM6SF2-interacting proteins by TAP coupled with mass spectrometry. TM6SF2 regulates APOB metabolism by binding and stabilizing APOB. ERLIN1 and ERLIN2 also promote APOB stabilization through stabilizing TM6SF2. Our study suggests that defective lipoprotein secretion, for example as a result of TM6SF2 E167K-induced APOB destabilization, underlies a class of NAFLD.

## Materials and methods

### Ethics statement

All procedures and care of animals were carried out in accordance with the guidelines and protocols approved by the Institutional Animal Care and Use Committee at the Wuhan University under protocol number WDSKY0201408.

### Reagents

Q5 high-fidelity DNA polymerase and T7E1 were from New England Biolabs. Bodipy (#3922) and colloidal blue staining kit (#LC6052) were from ThermoFisher Scientific. Oil Red O (#O0625) was from Sigma. Aspartate aminotransferase assay kit (#C010) was from Nanjing Jiancheng Bioengineering Institute. Total cholesterol assay kit and triglyceride assay kit were from Shanghai Kehua Bio-engineering. Hematoxylin and eosin (H&E) staining kit (#C0105) was from Beyotime.

### Plasmids

The coding region of mouse *Tm6sf2* gene was amplified from mouse liver and cloned into a p3×FLAG-TEV-2×Protein A-CMV-14 vector or a pcDNA3 vector containing a MYC epitope tag at the C terminus. The coding regions of *ERLIN1* and *ERLIN2* were amplified from Huh7 cells and cloned into a p3×FLAG-CMV-14 vector or a pcDNA3 vector containing a MYC epitope tag. TM6SF2 truncations were performed using a PCR-based strategy with KOD DNA polymerase (Takara).

### Cell culture

CRL1601 (McArdle RH7777 rat hepatoma cell), Huh7 and HEK293 cells were from ATCC and grown in Dulbecco’s modified Eagle’s medium supplemented with 100 units/mL penicillin, 100 μg/mL streptomycin sulfate and 10% fetal bovine serum (medium A) at 37°C with 5% CO_2_. CRL1601/TM6SF2-TAP stable cell line was generated by transiently transfecting CRL1601 cells with the TM6SF2-FLAG-TEV-Protein A construct using FuGENE HD (Promega). After 48 h, cells were switched to medium A supplemented with 200 μg/mL G418. Media were replaced every 2–3 days until single colonies were formed.

### Antibodies

Primary antibodies used for this study were as follows: rabbit anti-APOB (ab20737, Abcam); mouse anti-CHC (610500, BD Transduction Laboratories); rabbit anti-ERLIN1 (17311-1AP, proteintech); rabbit-anti ERLIN2 (14781-1-AP, proteintech); mouse anti-FLAG (F9291, Sigma); rabbit anti-FLAG (20543-1-AP, proteintech); mouse anti-MYC (sc-40, Santa Cruz Biotechnology); rabbit anti-MYC (06–549, Millipore); rabbit anti-TM6SF2 (AAS00444C, Antibody Verify). Horseradish peroxidase-conjugated goat anti-rabbit IgG (31460) and donkey anti-mouse IgG (715-035-150) antibodies were from Pierce and Jackson ImmunoResearch, respectively.

### Animals

All animal experiments were performed under the protocols approved by the Institutional Animal Care and Use Committee of Wuhan University. Eight-week-old BALB/c male mice were purchased from Hunan SJA Laboratory Animal Co. *Tm6sf2* heterozygous (*Tm6sf2*^*+/-*^) mice were generated by introducing a one-base deletion (67delC) in exon 1 using TALEN technique on a C57BL/6J background (Shanghai SIDANSAI Biotechnology). Male and female *Tm6sf2*^*+/-*^ mice were crossed to generate *Tm6sf2*^*-/-*^ mice and WT littermates. Mice were housed in a specific pathogen-free, temperature-controlled room with a 12-h light, 12-h dark-cycle. Mice were allowed *ad libitum* access to water and a standard laboratory diet (Beijing HFK Bioscience 1026, protein ≥18%, fat ≥4%, fiber ≤5%, ash ≤8%, moisture ≤10%, lysine ≥0.82%, calcium = 1.0%–1.8%, phosphorus = 0.6%–1.2%, and salt = 0.3%–0.8%). The feeding periods were indicated in the figure legends.

### Tandem affinity purification

CRL1601/TM6SF2-TAP stable cells were set up at a density of 1 × 10^6^ per 10-cm dish. After 48 h, cells were harvested and lysed in 1 mL of buffer A (1 × PBS, 5 mM EDTA, 5 mM EGTA,1% digitonin, plus protease inhibitor cocktail composed of 10 μM MG132, 10 μg/mL leupeptin, 5 μg/mL pepstatin A, 25 μg/mL ALLN and 1 mM PMSF), followed by preclear with protein A/G beads. Supernatants were incubated with IgG beads (sc-2003, Santa Cruz Biotechnology) at 4°C for 2 h. Beads were washed with buffer A five times and incubated with the TEV cutting buffer (PBS, 1% digitonin, 5 mM EDTA, 5 mM EGTA, 0.2 μg/μL TEV protease with 5 × protease inhibitors) at room temperature for 1 h. After centrifugation at 1,000 × *g* for 5 min, supernatants were collected and incubated with the anti-FLAG beads at 4°C for 2 h. Beads were washed five times with buffer A, and bound proteins were eluted using the FLAG peptides. Proteins were then subjected to mass spectrometry analysis using the Orbitrap analyzer (ThermoFisher Scientific).

### RNA Interference

Duplexes of siRNA were synthesized by Genepharma. The siRNA targeting human *TM6SF2*, *APOB*, *ERLIN1*, *ERLIN2* are as follows: 5’-GGTCTACAGCTTGTCCCAT-3’, 5’-GCATGTGGCTGGTAACCTA-3’; 5’-GCTCTGCAGAAAGACTTAA-3’; 5’-GCCCTGGTTTCCATCTCAT-3’, respectively. Transfection of siRNAs was carried out as previously described [41].

### Quantitative real-time PCR

Total RNA was extracted from cells or mouse livers using Trizol (T9424, Sigma). The equal amounts of RNA from the same treatment were pooled for cDNA synthesis with oligo dT and reverse transcriptase MLV (Promega). Gene expression was analyzed by quantitative real-time PCR on a BioRad CFX96 Real-Time System.

### Oil Red O staining

Oil Red O was dissolved in propylene glycol at a working concentration of 0.5% (w/v). Cells were grown on glass coverslips and fixed with 4% paraformaldehyde in PBS at room temperature for 30 min. After PBS wash, cells were stained in Oil Red O solution for 20 min. Cells were washed thoroughly under the running tap water and counterstained with hematoxylin for 10 s. Cells were examined and imaged under an Olympus BX53 microscope. Images were analyzed using ImageJ software (National Institutes of Health). The average area of Oil Red O-stained LDs was normalized by the number of cells in the same field.

### Immunofluorescence

Cells were grown on glass coverslips and fixed with 4% paraformaldehyde at room temperature for 20 min. After PBS wash, cells were permeabilized with 0.1% digitonin in PBS for 5 min and blocked in 10% fetal bovine serum in PBS for 1 h. Cells were then incubated with primary antibodies for 1 h at room temperature, washed with PBS three times and incubated with secondary antibodies for 1 h. For LD analysis, cells were stained with BODIPY 493/503 for 15 min and then DAPI for 5 min. Cells were examined and imaged under a Leica SP8 confocal microscope. The average area of BODIPY stained-LDs was normalized by the residing cell area.

### Immunoprecipitation

Triplicates of cells for each treatment were harvested and lysed in the immunoprecipitation buffer (1 × PBS, 0.1% NP-40 plus protease inhibitors). Whole cell lysates were incubated with anti-MYC or anti-FLAG beads at 4°C for 2 h. Beads were washed five times with the immunoprecipitation buffer, and the bound proteins were eluted in the 4 × loading buffer (150 mM Tris-HCl, pH 6.8, 12% SDS, 30% glycerol, 6% 2-mercaptoethanol, and 0.02% bromophenol blue) at 95°C for 10 min.

### Immunoblotting

Cells were harvested and lyzed with 120 μL of RIPA buffer supplemented with protease inhibitors. The protein concentration of lysates was determined using the BCA kit (ThermoFisher Scientific). Lysates were mixed with the membrane protein solubilization buffer (62.5 mM Tris-HCl, pH 6.8, 15% SDS, 8 M urea, 10% glycerol, and 100 mM DTT) plus the 4 × loading buffer and incubated at 37°C for 30 min. Proteins were resolved by SDS-PAGE and transferred to PVDF membrane. Blots were blocked with 5% bovine serum albumin in TBS plus 0.075% Tween (TBST) and probed with primary antibodies overnight at 4°C. After TBST wash, blots were incubated with secondary antibodies for 1 h at room temperature.

### AAV8-mediated knockdown

The shRNAs targeting mouse *Tm6sf2* (5’-CCGGGTGGCCTACCCAAAGTTGCCTCGAGGCAACTTTGGGTAGGCCACTTTTTG-3’), *Erlin1* (5’- CCGGGGTGGAGTCATGATCTATATTCTCGAGAATATAGATCATGACTCCACCTTTTTG-3’), *Erlin2* (5’- CCGGGGTCCCAAATGCAGTGTATGACTCGAGTCATACACTGCATTTGGGACCTTTTTG-3’) were inserted into the pLKO.1 vector followed by recombination with pAAV8-CAG-GFP. Adenovirus was packaged in HEK293 cells and purified with CsCl ultracentrifugation. Virus was tittered and administrated via caudal vein injection at a dose of 1 × 10^11^ viral genome per mouse. One week after injection, serum and livers were collected for analysis.

### Serum and liver chemistry

Mice were fasted for 4 h before sacrifice. Serum was prepared by centrifuging blood at 1,500 × g for 10 min. Forty milligrams of liver were harvested and homogenized three times in a mixture of chloroform and methanol (2:1, v/v) using Precellys 24 (Bertin) at 5,500 rpm for 10 s each. Extracts were shaken at 1,000 × g for 1 h at 37°C and centrifuged at 2,000 × g for 10 min. Supernatants were collected, mixed with double-distilled H_2_O, followed by centrifugation at 2,000 × g for 10 min. The organic phase was dried under N_2_ and then reconstituted in ethanol. Total cholesterol and triglyceride levels in the serum and liver were determined using the cholesterol assay kit and triglyceride assay kit [42].

### Hematoxylin and eosin staining

Liver samples were fixed in 4% paraformaldehyde for 24 h and placed in 30% sucrose solution overnight. After dehydration, samples were embedded in OCT compound and cut into 10 μm-thick sections. Sections were wash by double-distilled H_2_O for 2 min and stained with the hematoxylin and eosin staining kit [43]. Sections were examined and imaged under an Olympus BX53 microscope.

## Supporting information

S1 Fig Identification of TM6SF2-interacting proteins using tandem affinity purification (TAP) coupled with tandem mass spectrometry (MS). (A) Schematic of the construct expressing TM6SF2-3×FLAG-tobacco etch virus (TEV) cleavage site-2×Protein A fusion protein (TM6SF2-TAP). (B) CRL1601 cells were transfected with the plasmid encoding TM6SF2-TAP. After 48 h, cells were fixed, with lipid droplets stained with Bodipy (green) and the transiently expressed TM6SF2-TAP with the anti-FLAG antibody (Red). Nuclei were counterstained with DAPI (blue). Scale bars, 25 μm (main), 10 μm (inset). Cell overexpressing the transfected protein (o/e) and neighboring un-transfected one (yellow star) are indicated. (C) Immunoblotting analysis showing robust expression of TM6SF2 in the CRL1601/TM6SF2-TAP stable cells compared with the parental CRL1601 cells. (D) Strategy for identifying TM6SF2-interacting proteins using TAP-MS. (E) Colloidal blue staining showing the purified TM6SF2-TAP protein. (F) List of TM6SF2-interacting proteins identified from MS analysis from high to low abundance.
(TIF)

S2 Fig Knockdown efficiency of shRNA in various tissues. Eight-week-old, chow-fed BALB/c male mice were injected with 1 × 10^11^ AAV expressing control shRNA (AAV-shCtr) or shRNA targeting *Tm6sf2* (AAV-sh*Tm6sf2*) (n = 5 per group, the same cohorts as used in Fig 7). After 2 weeks, mice were sacrificed after a 4-h fast and subjected to quantitative real-time PCR analysis. The mean levels of *Tm6sf2* transcript in each tissue of mice receiving AAV-shCtr were set to 1. Data are presented as mean±SD. Student’s *t*-test. ***P<0.001, NS, no significance. BAT, brown adipose tissue; E-WAT, epididymal white adipose tissue; I-WAT, inguinal white adipose tissue; SI, small intestine.
(TIF)

S3 Fig Time-of-day effect of TM6SF2-mediated APOB stabilization. Eight-week-old, chow-fed male *Tm6sf2*^*+/+*^ and *Tm6sf2*^*-/-*^ mice (n = 3 per group) were deprived of food at 7:00 a.m. for 4 h (Procedure #1), or at 10:00 a.m. for 4 h (Procedure #2), and then sacrificed for various assays. (A) Representative blots showing the expression of APOB and TM6SF2 in the liver. Clathrin heavy chain (CHC) was used as a loading control. Densitometry of APOB48 and APOB100 was normalized to that of CHC. Data are presented as mean±SD. (B) Serum total cholesterol (TC) and triglyceride (TG) levels. (C) Liver TC and TG levels. Data are presented as mean±SD. Student’s *t*-test. *P<0.05, **P<0.01, NS, no significance.
(TIF)
