# Supplementary figures and images for: Disruption of the ERLIN–TM6SF2–APOB complex destabilizes APOB and contributes to non-alcoholic fatty liver disease
Source: PLoS Genet. 2020 Aug 10;16(8):e1008955. doi: 10.1371/journal.pgen.1008955 (PMC7462549; doi:10.1371/journal.pgen.1008955)

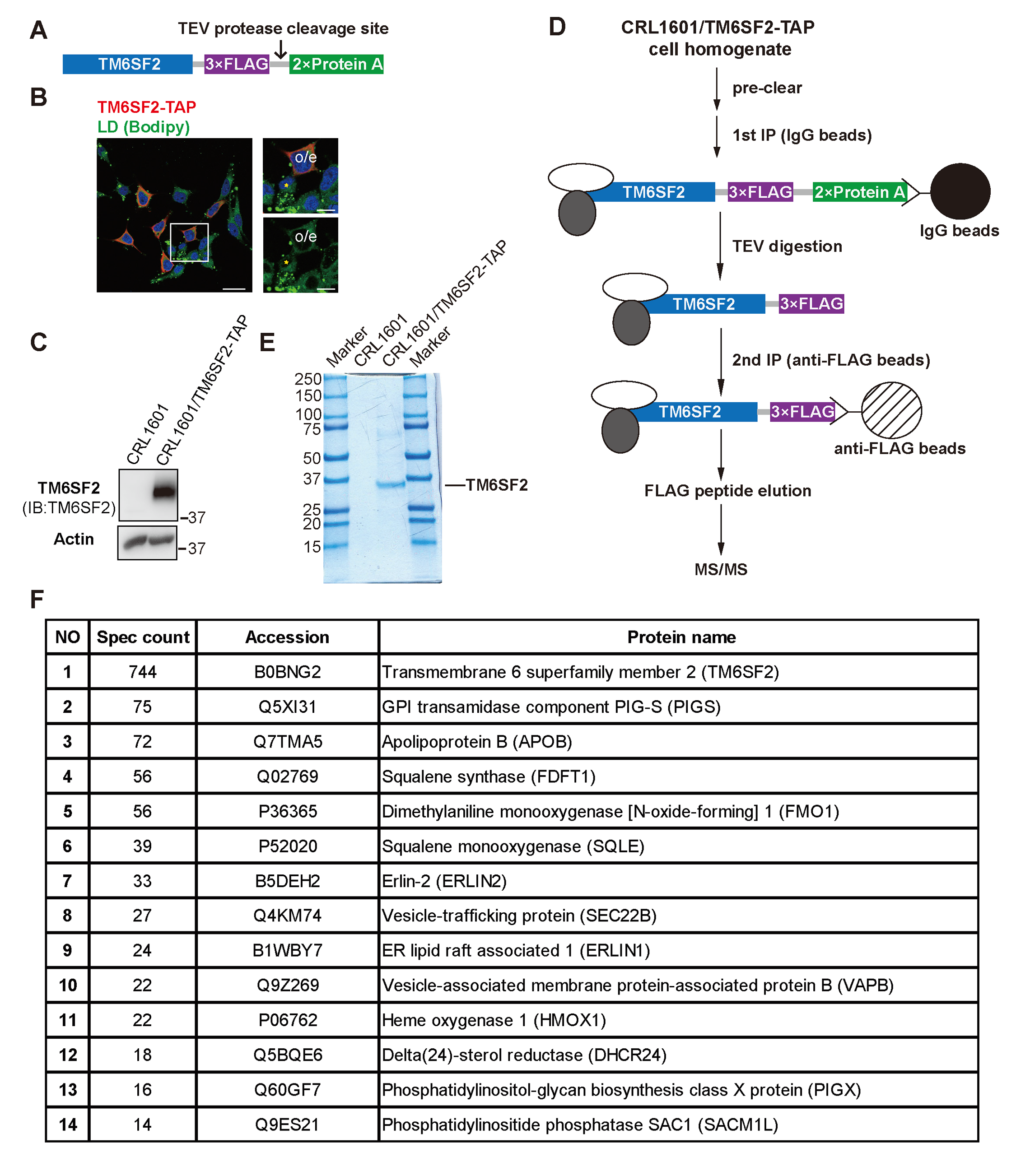

Supplement: S1 Fig — (A) Schematic of the construct expressing TM6SF2-3×FLAG-tobacco etch virus (TEV) cleavage site-2×Protein A fusion protein (TM6SF2-TAP). (B) CRL1601 cells were transfected with the plasmid encoding TM6SF2-TAP. After 48 h, cells were fixed, with lipid droplets stained with Bodipy (green) and the transiently expressed TM6SF2-TAP with the anti-FLAG antibody (Red). Nuclei were counterstained with DAPI (blue). Scale bars, 25 μm (main), 10 μm (inset). Cell overexpressing the transfected protein (o/e) and neighboring un-transfected one (yellow star) are indicated. (C) Immunoblotting analysis showing robust expression of TM6SF2 in the CRL1601/TM6SF2-TAP stable cells compared with the parental CRL1601 cells. (D) Strategy for identifying TM6SF2-interacting proteins using TAP-MS. (E) Colloidal blue staining showing the purified TM6SF2-TAP protein. (F) List of TM6SF2-interacting proteins identified from MS analysis from high to low abundance. (TIF) [file pgen.1008955.s001.tif]

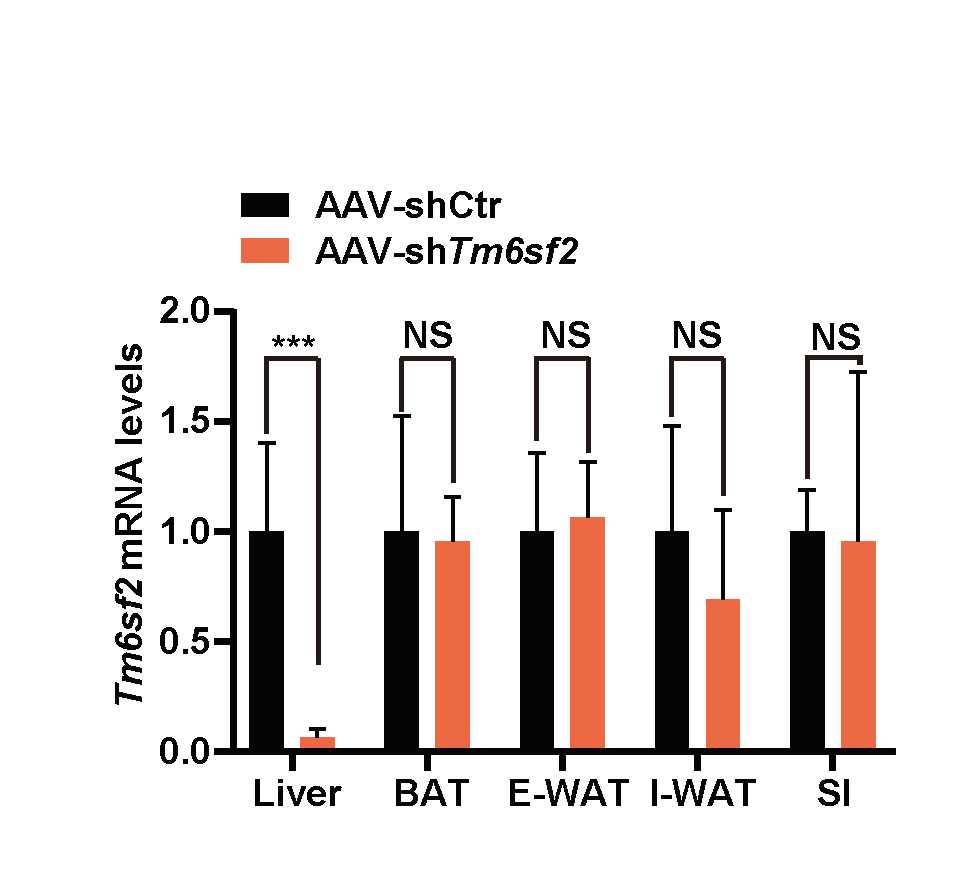

Supplement: S2 Fig — Eight-week-old, chow-fed BALB/c male mice were injected with 1 × 10^11^ AAV expressing control shRNA (AAV-shCtr) or shRNA targeting Tm6sf2 (AAV-shTm6sf2) (n = 5 per group, the same cohorts as used in Fig 7). After 2 weeks, mice were sacrificed after a 4-h fast and subjected to quantitative real-time PCR analysis. The mean levels of Tm6sf2 transcript in each tissue of mice receiving AAV-shCtr were set to 1. Data are presented as mean±SD. Student’s t-test. ***P<0.001, NS, no significance. BAT, brown adipose tissue; E-WAT, epididymal white adipose tissue; I-WAT, inguinal white adipose tissue; SI, small intestine. (TIF) [file pgen.1008955.s002.tif]

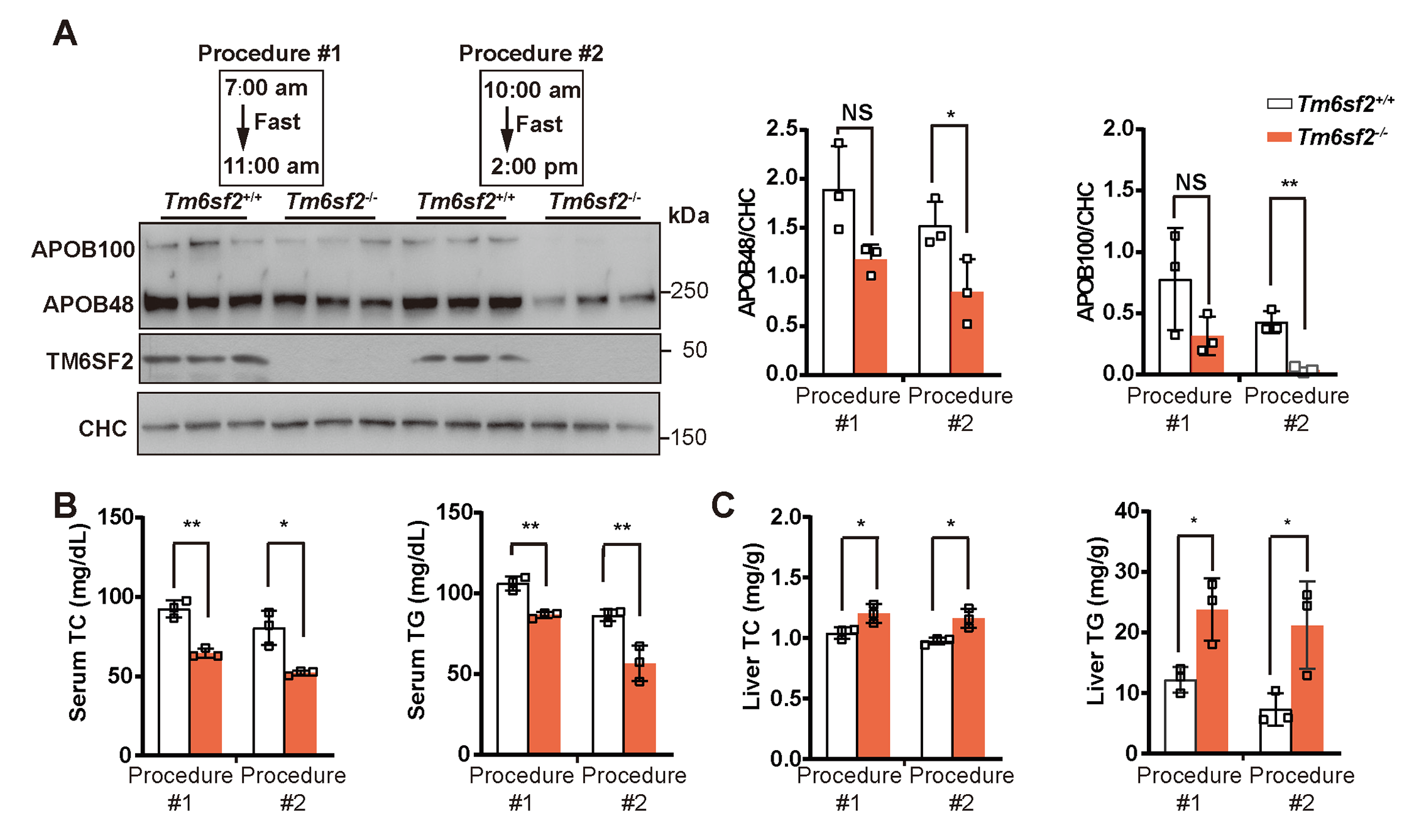

Supplement: S3 Fig — Eight-week-old, chow-fed male Tm6sf2+/+ and Tm6sf2-/- mice (n = 3 per group) were deprived of food at 7:00 a.m. for 4 h (Procedure #1), or at 10:00 a.m. for 4 h (Procedure #2), and then sacrificed for various assays. (A) Representative blots showing the expression of APOB and TM6SF2 in the liver. Clathrin heavy chain (CHC) was used as a loading control. Densitometry of APOB48 and APOB100 was normalized to that of CHC. Data are presented as mean±SD. (B) Serum total cholesterol (TC) and triglyceride (TG) levels. (C) Liver TC and TG levels. Data are presented as mean±SD. Student’s t-test. *P<0.05, **P<0.01, NS, no significance. (TIF) [file pgen.1008955.s003.tif]
